# Increased use of hypnotics in individuals with celiac disease: a nationwide case-control study

**DOI:** 10.1186/s12876-015-0236-z

**Published:** 2015-02-05

**Authors:** Karl Mårild, Timothy I Morgenthaler, Virend K Somers, Suresh Kotagal, Joseph A Murray, Jonas F Ludvigsson

**Affiliations:** 1 Department of Medical Epidemiology and Biostatistics, Karolinska Institutet, Stockholm, Sweden; 2 Division of Epidemiology, Norwegian Institute of Public Health, Oslo, Norway; 3 Division of Pulmonary and Critical Care Medicine, Center for Sleep Medicine, Mayo Clinic, Rochester, MN USA; 4 Department of Internal Medicine, Mayo Clinic, Rochester, MN USA; 5 Departments of Neurology and Pediatrics, The Center for Sleep Medicine, Mayo Clinic, Rochester, MN USA; 6 Departments of Medicine and Immunology, Mayo Clinic, Rochester, MN USA; 7 Department of Pediatrics, Örebro University Hospital, Örebro, Sweden; 8 Astrid Lindgren Children’s Hospital, Stockholm, Sweden

**Keywords:** Coeliac, Immune-mediated, Insomnia, Sleep disorders, Small intestine

## Abstract

**Background:**

Although poor sleep is common in numerous gastrointestinal diseases, data are scarce on the risk of poor sleep in celiac disease. The objective of this study was to estimate the risk of repeated use of hypnotics among individuals with celiac disease as a proxy measure for poor sleep.

**Methods:**

This is a nationwide case–control study including 2933 individuals with celiac disease and 14,571 matched controls from the general Swedish population. Poor sleep was defined as ≥2 prescriptions of hypnotics using prospective data from the National Prescribed Drug Register (data capture: July 2005-January 2008). We estimated odds ratios and hazard ratios for poor sleep before and after celiac disease diagnosis respectively.

**Results:**

In this study, poor sleep was seen in 129/2933 individuals (4.4%) with celiac disease, as compared with 487/14,571 controls (3.3%) (odds ratio = 1.33; 95% CI = 1.08-1.62). Data restricted to sleep complaints starting ≥1 year before celiac disease diagnosis revealed largely unchanged risk estimates (odds ratio = 1.23; 95% CI = 0.88-1.71) as compared with the overall risk (odds ratio 1.33). The risk of poor sleep in celiac disease was essentially not influenced by adjustment for concomitant psychiatric comorbidity (n = 1744, adjusted odds ratio =1.26; 95% CI = 1.02-1.54) or restless legs syndrome (n = 108, adjusted odds ratio = 1.33; 95% CI = 1.08-1.63). Poor sleep was also more common after celiac disease diagnosis as compared with matched controls (hazard ratio = 1.36; 95% CI = 1.30-1.41).

**Conclusions:**

In conclusion, individuals with celiac disease suffer an increased risk of poor sleep, both before and after diagnosis. Although we cannot rule out that surveillance bias has contributed to our findings, our results are consistent with previous data suggesting that sleep complaints may be a manifestation of celiac disease.

**Electronic supplementary material:**

The online version of this article (doi:10.1186/s12876-015-0236-z) contains supplementary material, which is available to authorized users.

## Background

Celiac disease (CD) is a chronic autoimmune disorder affecting 1-2% of the Western population [1]. In CD, dietary gluten not only leads to small-intestinal villous atrophy, but also affects organs outside the gastrointestinal tract [2]. Individuals with CD therefore present with a great variety of symptoms and signs: classical symptoms of malabsorption (e.g. diarrhea with abdominal pain) and non-classical symptoms (e.g. fatigue, peripheral neuropathy and restless legs syndrome (RLS) [3]).

Poor sleep commonly includes difficulties in initiating and maintaining sleep, or a non-restorative sleep with or without daytime consequences [4]. According to Swedish data, up to 20% of those with a self-reported sleeping disorder use prescribed sleeping pills [5]. Although prescription of hypnotics, such as benzodiazepines or benzodiazepine-related drugs, is primarily recommended for short-term treatment of acute sleep disorders, in routine clinical practice pharmacological treatment is also common for prolonged-periods (months to years) of poor sleep [6].

Poor sleep frequently occurs in a number of gastrointestinal diseases, e.g. inflammatory bowel disease (IBD) [7]. Not only may gastrointestinal symptoms impair the quality of sleep, but poor sleep may also have a detrimental effect on the course of both functional and inflammatory gastrointestinal conditions [7]. Poor sleep may also add to the burden of disease by reducing the quality of life [8] and predispose to the development of comorbidities, such as psychiatric diseases [9]. Although poor sleep has been closely studied in many other gastrointestinal diseases [10], to our knowledge, only one previous study has addressed the risk of poor sleep in CD [11]. That cross-sectional study, based on 60 patients identified at one tertiary center, found poorer sleep quality among individuals with CD, both before and after treatment with a gluten-free diet, as compared with healthy volunteers [11].

In the current nationwide study including some 2900 individuals with CD we examined the risk of poor sleep defined as repeated use of hypnotics, both before and after CD diagnosis.

## Methods

Data on individuals with CD were linked to the Swedish Prescribed Drug Register [12] in order to examine the risk of poor sleep in CD. Poor sleep was defined as repeated use (≥2 prescriptions) of hypnotics.

### Study population

Between 2006 and 2008, we searched the computerized register from all Sweden’s pathology departments (n = 28) to identify individuals with biopsy-verified CD, defined as small-intestinal villous atrophy (Marsh grade 3) [13]. We have earlier shown that 95% of Swedish individuals with villous atrophy have CD [13]. The current study was based on the same dataset as described in our study on mortality in CD, [14] originally including 29,096 individuals with CD diagnosed between 1969 and 2008. Time of CD diagnosis was defined as time of first biopsy showing villous atrophy. For each of these individuals with CD the government agency Statistics Sweden tried to identify five controls matched for age, sex, calendar period of birth and county of residence through the Total Population Register, which includes demographic data on all Swedish residents [15]. Controls were sampled from all Swedish residents in whom the computerized pathology registers did not indicate prior duodenal/jejunal biopsy. After exclusion of controls with data irregularities and those that could not be matched 144,522 controls were identified. Due to lack of eligible controls some individuals with celiac disease only had four controls and the average numb of controls per individual with celiac disease was slightly below 5. Where a patient had celiac disease, especially if diagnosed in old age, the responsible government agency sometimes failed to identify 5 matching controls. E.g. 76-year old man diagnosed with celiac disease in 1989 in Dalsland county. This means that this person was born in 1913. It may then have been that in 1989 there were only 4 other males aged 76 year in the part of Dalsland where this man lived.

**Figure 1:**
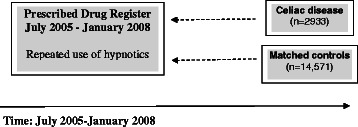
**Overview methodology of main analysis: risk of poor sleep before celiac disease diagnosis.**

**Figure 2:**
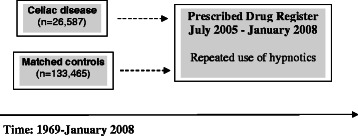
**Overview methodology of subanalysis: risk of poor sleep after celiac disease diagnosis.**

We then linked data on individuals with CD and their matched controls to the Prescribed Drug Register [12] (established July 2005) in order to identify individuals with repeated use of hypnotics before CD diagnosis (and corresponding date in controls). Our main analysis was therefore restricted to individuals diagnosed with CD between July 2005 and January 2008, including 2,933 individuals with CD and 14,571 matched controls. In order to estimate the risk of poor sleep *after* CD diagnosis, we performed a pre-planned subanalysis including 26,587 individuals with CD diagnosed between 1969 and 2008 and 133,465 matched controls with a follow-up until July 2005 (start of the Prescribed Drug Register) and with no use of hypnotics prior to CD diagnosis. See Figures 1 and 2 for overview of study methodology.

### Poor sleep

The Prescribed Drug Register, established in July 2005, contains prospectively recorded high-quality data on virtually all prescribed drugs in Sweden [12]. In Sweden, the use of hypnotics is strictly controlled and sleep medications are generally not sold over the counter.

We collected data on use of hypnotics from July 1st 2005 through January 29th 2008 and up to the date of CD diagnosis (and the corresponding date in matched controls). In order to minimize the risk of false positive cases we restricted our definition of poor sleep as ≥2 prescriptions of hypnotics before CD diagnosis. Hypnotics were classified according to the Anatomical Therapeutic Chemical (ATC) pharmaceutical classification system [16]: benzodiazepine-related drugs (i.e. non-benzodiazepine GABA-A receptor agonists), benzodiazepines, other hypnotics and melatonin receptor agonists (see Additional file 1). A similar classification of hypnotics has previously been used [17]. Regrettably, the National Patient Register is unsuitable for identification of poor sleep (e.g. diagnosed insomnia) as this registry is limited to International Classification of Disease (ICD) codes from inpatient and non-primary outpatient care clinics with low sensitivity to ascertain poor sleep.

### Post-hoc analyses

To investigate potential contributing factors to poor sleep in individuals with CD we performed a post-hoc analysis adjusting for sleep apnea and RLS since these two conditions may impair sleep [18-20]. Most studies have indicated an increased risk of RLS in CD [3,21], possibly due to prevalent iron deficiency in celiac patients. There are no data on the specific risk of sleep apnea in individuals with CD. We identified RLS using relevant ICD-codes in the National Patient Register (ICD-8: 787.00; ICD-9: 333X and ICD-10: G25.8) or as prescription of dopaminergic drugs typically used in the treatment of RLS (excluding individuals with concomitant Parkinson’s disease) (see Additional file 2 for ATC-codes). Sleep apnea was identified using the National Patient Register (ICD-10: G47.3).

### Statistical analyses

We used conditional logistic regression to estimate Odds Ratios (ORs) and 95% confidence intervals (CIs). Through conditional logistic regression each stratum (one individual with CD compared with his/her matched controls) was analyzed separately before a summary OR was calculated [22]. This statistical approach eliminated the effect of sex, age, county and calendar year on our ORs. Practically, conditional logistic regression works similar as regular logistic regression except its comparison is carried out in matched sets.

We performed stratified analyses by age at CD diagnosis (<19 years, 19–39 years, 40–59 years and ≥60 years) and sex. The therapeutic indications differ between types of hypnotics. In pre-planned subanalyses we therefore estimated the association between CD and poor sleep according to the type of hypnotics used: benzodiazepine-related drugs, benzodiazepines, other hypnotics and melatonin receptor agonists. We also estimated the dose- and time-dependent association between use of hypnotics and subsequent CD: (1) when individuals had received two or at least three prescriptions of hypnotics and (2) when hypnotics had been prescribed in the last year (≤365 days) or more than one year (>365 days) before CD diagnosis, respectively.

We considered education level [4,23], as a measure of socioeconomic position, psychiatric comorbidity [24] and epilepsy [25] as potential confounders. In a subanalysis we therefore adjusted for education level using seven predefined education categories determined by Statistics Sweden. We also adjusted for epilepsy and psychiatric comorbidity, including substance use and mood disorders, using relevant ICD-codes recorded in the National Patient Register [26] (see Additional file 3 for ICD-codes).

### Subanalyses

We used Cox regression to estimate Hazard Ratios (HRs) and 95% CIs for poor sleep (≥2 prescriptions of hypnotics) *after* CD diagnosis. In this subanalysis we included 26,587 individuals with CD diagnosed between 1969 and 2008 and 133,465 matched controls with a follow-up until July 2005 (start of the Prescribed Drug Register) and with no use of hypnotics prior to CD diagnosis (see Figure 2 for overview of methodology of subanalysis). Follow-up started at time of CD diagnosis (and corresponding date in controls) and ended at time of first prescription of hypnotics (July 2005 or later), death, emigration or on December 31st 2009, whichever occurred first. In controls, follow-up could also end if the individual was diagnosed with CD. We performed stratified analyses by sex and age at CD diagnosis as well as subanalyses adjusting for education level, psychiatric comorbidity and epilepsy.

SPSS version 20.0 was used for all statistical analyses.

### Ethics

This study was conducted in accordance with the national and institutional standards and was approved by the Regional Ethical Vetting Board in Stockholm. Since none of the participants was contacted and individual information was anonymized prior to the analyses, informed consent was not required by the research ethics committee.

## Results

Some 40% of the individuals with CD had been diagnosed in childhood and 19% were aged 60 years or more at time of diagnosis (median age at CD diagnosis was 28 years). Most of the individuals with CD included in our main analysis were diagnosed in year 2006 and some 60% of the study participants were female (Table 1).

**Table 1:** **Descriptive characteristics of individuals with celiac disease (CD) and matched controls**

	Celiac disease	Controls
**Total**	2933	14,571
**Sex**		
Females (%)	1796 (61.2)	8926 (61.3)
Males (%)	1137 (38.8)	5645 (38.7)
**Age at study entry, years (median; range** ^**a**^ **)**	28; 0-94	28; 0-94
**Age groups**		
≤19 years (%)	1218 (41.5)	6074 (41.7)
20-39 years (%)	566 (19.3)	2809 (19.3)
40-59 years (%)	583 (19.9)	2905 (19.9)
≥60 years (%)	566 (19.3)	2783 (19.1)
**Calendar year of CD diagnosis**		
2005^b^ (%)	819 (27.9)	4062 (27.9)
2006 (%)	1828 (62.3)	9097 (62.4)
2007^c^ (%)	274 (9.3)	1352 (9.3)
2008^d^ (%)	12 (0.4)	60 (0.4)

^a^Ten children were diagnosed of CD during their first year of life and are in the table referred to as age “0”.

^b^Beginning of study period: July 1st 2005.

^c^Most pathology departments only delivered small-intestinal biopsy data up to the beginning of 2007. Therefore this study includes fewer individuals with CD diagnosed 2007–2008, as compared with 2005–2006.

^d^End of study period: January 29th 2008.

Overall, 129 individuals (4.4%) with CD had been treated for poor sleep, as compared with 487 matched controls (3.3%), corresponding to an OR of 1.33 (95% CI = 1.08-1.62) (adjustment for education level: adjusted OR = 1.34; 95% CI = 1.09-1.64). Because specific hypnotics may occasionally also be used in the treatment of epilepsy and various psychiatric diseases we adjusted for these comorbidities although with only a minor change in risk estimates (epilepsy: n = 207; psychiatric disease: n = 1744; adjusted OR = 1.26; 95% CI = 1.02-1.54).

The prevalence of poor sleep treated with prescribed hypnotics increased with increasing age at CD diagnosis being most common in those aged 60 years or older; and there were very few children treated for poor sleep (Table 2). Poor sleep was significantly associated with CD in individuals aged ≥60 years at time of diagnosis (OR = 1.40; 95% CI = 1.07-1.83); however the risk estimates did not attain statistical significance in the younger age groups and with overlapping CIs between age groups. Overall, poor sleep was more prevalent in females than in males (Table 2). However, there were only small differences in risk estimates for poor sleep between females (OR = 1.38; 95% CI = 1.08-1.76) and males with CD (OR = 1.21; 95% CI = 0.83-1.75) as compared with sex-matched controls.

**Table 2:** **Risk of poor sleep before celiac disease diagnosis**

	Celiac disease (%)	Controls (%)	Odds ratio	95% CI
**Poor sleep** ^**a**^	128/2933 (4.4)	487/14,571 (3.3)	1.33	1.08-1.62
**Type of hypnotics used** ^**b**^				
Benzodiazepine-related drugs	111 (3.8)	380 (2.6)	1.47	1.18-1.82
Benzodiazepines	17 (0.6)	65 (0.4)	1.28	0.76-2.16
Other hypnotics	32 (1.1)	143 (1.0)	1.11	0.76-1.62
Melatonin receptor agonists^c^	0 (0.0)	2 (0.0)	-	-
**≥3 prescriptions of hypnotics** ^**c**^				
Any hypnotics	78 (2.7)	359 (2.5)	1.07	0.84-1.38
Benzodiazepine-related drugs	62 (2.1)	256 (1.8)	1.19	0.90-1.58
Benzodiazepines	7 (0.2)	41 (0.3)	0.85	0.39-1.88
Other hypnotics	12 (0.4)	86 (0.6)	0.70	0.39-1.28
Melatonin receptor agonists^d^	0 (0.0)	2 (0.0)	-	-
**Age**				
≤19 years	1/1218 (0.1)	4/6074 (0.1)	1.23	0.15-9.80
20-39 years	8/566 (1.4)	49/2809 (1.7)	0.82	0.39-1.72
40-59 years	39/583 (6.7)	142/2905 (4.9)	1.38	0.96-1.97
≥60 years	80/566 (14.1)	292/2783 (10.5)	1.40	1.07-1.83
**Sex**				
Males	36/1137 (3.2)	150/5645 (2.7)	1.21	0.83-1.75
Females	92/1796 (5.1)	337/8926 (3.8)	1.38	1.08-1.76

Odds ratios estimated through conditional logistic regression modelling.

^a^Poor sleep defined as ≥2 prescriptions of hypnotics before celiac disease diagnosis. Data capture of hypnotics: July 1st 2005-January 29th 2008.

^b^See Additional file 1: Table S1 for anatomical therapeutic chemical (ATC) codes used to classify hypnotics.

^c^The category “**≥**3 prescriptions of hypnotics” is being compared with the category “<3 prescriptions of hypnotics”.

^d^Due to lack of events no odds ratio was estimated.

Benzodiazepine-related drugs (e.g. Zolpidem) were the most frequently used type of hypnotics, being used by 3.8% of individuals with CD and 2.6% of controls, corresponding to an OR of 1.47 (95% CI = 1.18-1.82). There were no significant associations between CD and use of any of the remaining types of hypnotics (Table 2). We found no indication of a dose–response relationship, with similar risk estimates among individuals with at least two prescriptions of hypnotics, as among those with three or more prescriptions of hypnotics (Table 2).

To estimate a possible time-dependent association between poor sleep and subsequent CD we performed two subanalyses estimating the association between poor sleep present in the last year (≤365 days) and poor sleep present more than one year (≥366 days) before CD diagnosis. Some 4.3% of individuals with CD, as compared with some 3.2% in controls, suffered from poor sleep in the year before CD diagnosis (OR = 1.35; 95% CI = 1.10-1.65). The OR remained largely unchanged when we restricted our data to poor sleep present more than one year before CD diagnosis (OR = 1.23; 95% CI = 0.88-1.71). In Tables 3 and 4 we describe the association between CD and poor sleep according to used hypnotics subgroup and according to time to CD diagnosis.

**Table 3:** **Risk of poor sleep starting within 1 year before celiac disease diagnosis**

	Celiac disease n = 2,933 (%)	Controls n = 14,571 (%)	Odds ratio	95% CI
**Poor sleep** ^**a**^	126 (4.3)	471 (3.2)	1.35	1.10-1.65
**Type of hypnotics used** ^**b**^				
Benzodiazepine-related drugs	84 (2.9)	281 (1.9)	1.48	1.16-1.90
Benzodiazepines	5 (0.2)	35 (0.2)	0.71	0.28-1.79
Other hypnotics	7 (0.2)	56 (0.4)	0.63	0.29-1.37
Melatonin receptor agonists^c^	0 (0.0)	1 (0.0)	-	-

Odds ratios estimated through conditional logistic regression modelling.

^a^Poor sleep defined as ≥2 prescriptions of hypnotics before celiac disease diagnosis. Data capture of hypnotics: July 1st 2005-January 29th 2008.

^b^See Additional file 1: Table S1 for anatomical therapeutic chemical (ATC) codes used to classify hypnotics.

^c^Due to lack of events no odds ratio was estimated.

**Table 4:** **Risk of poor sleep starting more than 1 year before celiac disease diagnosis**

	Celiac disease n = 1086 (%)	Controls n = 5382 (%)	Odds ratio	95% CI
**Poor sleep** ^**a**^	49 (4.5)	200 (3.7)	1.23	0.88-1.71
**Type of hypnotics used** ^**b**^				
Benzodiazepine-related drugs	40 (3.7)	151 (2.8)	1.32	0.92-1.90
Benzodiazepines	7 (0.6)	20 (0.4)	1.70	0.73-3.81
Other hypnotics	5 (0.5)	51 (0.9)	0.49	0.20-1.23
Melatonin receptor agonists^c^	-	-	-	-

^a^Poor sleep defined as ≥2 prescriptions of hypnotics before celiac disease diagnosis. Data capture of hypnotics: July 1st 2006-January 29th 2008.

^b^See Additional file 1: Table S1 for anatomical therapeutic chemical (ATC) codes used to classify hypnotics.

^c^Due to lack of events no odds ratio was estimated.

In a number of pre-planned subanalyses we estimated the risk of poor sleep after CD diagnosis (and corresponding date in controls). During follow-up, 3323 of 26,587 individuals (12.5%) were treated for poor sleep after CD diagnosis, as compared with 13,067/133,465 (9.8%) among controls, corresponding to a HR of 1.36 (95% CI = 1.30-1.41). The increased risk of poor sleep after CD diagnosis remained largely unchanged when adjusted for education level, epilepsy or psychiatric comorbidity (adjusted HR = 1.30; 95% CI = 1.25-1.36). Furthermore, individuals with CD had a similar increased risk poor sleep in the future irrespective of age at CD diagnosis, sex and according to type of hypnotic used (Table 5).

**Table 5:** **Risk of poor sleep after celiac disease diagnosis**

	Celiac disease n = 26,587 (%)	Controls n = 133,465 (%)	Hazard ratio	95% CI
**Poor sleep** ^**a**^	3323 (12.5)	13,067 (9.8)	1.36	1.30-1.41
**Age at CD diagnosis**				
≤19 years	298/11,708 (2.5)	1058/57,940 (1.8)	1.41	1.24-1.61
20-39 years	608/5170 (11.8)	2003/25,672 (7.8)	1.54	1.41-1.69
40-59 years	1223/5960 (20.5)	4659/30,252 (15.4)	1.37	1.29-1.47
≥60 years	1194/3749 (31.8)	5347/19,601 (27.3)	1.24	1.16-1.33
**Sex**				
Males	957/9815 (9.8)	3920/49,688 (7.9)	1.32	1.22-1.42
Females	2366/16,772 (14.1)	9147/83,777 (10.9)	1.37	1.31-1.44
**Type of hypnotics used** ^**b**^				
Benzodiazepine-related drugs	2782/26,591 (10.5)	10898/133,492 (8.2)	1.35	1.30-1.41
Benzodiazepines	394/26,610 (1.5)	1334/133,547 (1.0)	1.59	1.41-1.78
Other hypnotics	1191/26,604 (4.5)	4847/133,536 (3.6)	1.28	1.20-1.37
Melatonin receptor agonists	148/26,611 (0.6)	445/133,557 (0.3)	1.69	1.40-2.04

Hazard ratios estimated through cox regression modelling.

^a^Poor sleep defined as ≥2 prescriptions of hypnotics after CD diagnosis. Data capture of hypnotics: July 1st 2005-January 29th 2008.

^b^See Additional file 1: Table S1 for anatomical therapeutic chemical (ATC) codes used to classify hypnotics.

### Post-hoc analyses

To investigate definitive sleep disorders among individuals with CD and poor sleep we identified those with a previous diagnosis of sleep apnea or with RLS (defined by relevant ICD-code or prescription of drugs typically used in RLS). In 19/2933 individuals with CD (0.6%) a diagnosis of RLS was present, as compared with 89/14,571 (0.6%) in controls. Sleep apnea was identified in 10/2933 (0.3%) of individuals with CD and in 63/14571 (0.4%) of controls. Adjustment for sleep apnea and RLS did not influence the risk of poor sleep in CD (adjusted OR = 1.33; 95% CI = 1.08-1.63).

## Discussion

In this nationwide case–control study we found a modestly increased risk of poor sleep (≥2 prescriptions of hypnotics) in individuals with CD, both before and after diagnosis. This association was not influenced by adjustment for education, psychiatric disease, sleep apnea or RLS. Although, we cannot rule out that surveillance bias might have influenced our results, i.e. that individuals with poor sleep also had other complaints resulting in a CD investigation, the consistency of our results with previous data [11] suggests that sleep complaints may be a manifestation of CD.

### Strengths and weaknesses

A strength of our study is the use of prospectively recorded exposure and outcome data, eliminating the risk of recall bias. Further, the large statistical power allowed for important subanalyses, such as examination of dose- and time-dependent associations. We were also able to adjust for several potential confounders, e.g. psychiatric comorbidities.

The use of biopsy data to identify CD brings several advantages, such as being able to identify an average CD population. The use of biopsy data also minimizes the risk of CD misclassification. In Sweden, small intestinal biopsy has long been the gold standard for diagnosing CD and previous data have shown that more than 95% of Swedish physicians perform a small-intestinal biopsy before diagnosing CD [13]. An earlier validation study found that 95% of Swedish individuals with villous atrophy have CD [13].

This study has some limitations. Although it is likely that most of the individuals with repeated use of hypnotics suffer from some form of sleep complaints [4], such as difficulty initiating sleep, disrupted sleep and non-restorative sleep, we lack data on the specific nature of these complaints. Furthermore, previous data have shown that relatively few of those with a self-reported sleeping disorder receive treatment with hypnotics [5]. Although the pathogenesis may be similar in mild and more severe sleep complaints, our results may primarily represent severe sleep complaints requiring hypnotic prescription. Traditionally, prescription of hypnotics in children with poor sleep has been limited, and with the short follow-up of children in this study we may have underestimated the prevalence of poor sleep in children.

To minimize the risk of false positive cases we restricted our definition of poor sleep to repeated (≥2) prescriptions of drugs that are mainly indicated for the treatment of poor sleep. We acknowledge that some of the study participants with sleep complaints may have been prescribed treatments that were not included in our definition of hypnotics, e.g. sedating antidepressants. Furthermore, non-adherence to prescribed hypnotics as well as the left truncation of exposure data with no data on hypnotic use before July 2005, may also cause misclassification of our definition of poor sleep. However, such potential misclassification should not differ by CD status and therefore not lead to spurious associations, but might instead somewhat underestimate the true effect.

### Previous studies

We know of only one previous study on the risk of poor sleep in CD. In 2010, Zingone *et al.* [11] used the Pittsburg Sleep Quality Index and found that compared with healthy volunteers, both untreated and treated celiac patients had significantly poorer sleep quality, including prolonged sleep latency and shorter duration of sleep. In this study, including 60 patients from a tertiary centre, sleep quality was inversely associated with both mental and physical health, but was not associated with the intensity of gastrointestinal symptoms. Our results are consistent with the results of Zingone *et al.,* although the studies differ by study design.

Poor sleep has been recognized as a manifestation of several gastrointestinal conditions [10,27] including active as well as inactive IBD [7]. Conversely, poor sleep may have a detrimental effect on the course of both functional [28] and inflammatory gastrointestinal conditions, and several studies on IBD have found that sleep impairment may increase the risk of disease flares [29,30]. However, even though poor sleep has been shown to influence ubiquitous pro-inflammatory mediators [31] there is no plausible biological explanation for a causal association between poor sleep and development of CD. A non-causal association between CD and poor sleep is further supported by the lack of a dose- and time-dependent association in this study.

### Interpretation of findings

We found an increased risk of poor sleep both before and after CD diagnosis that was not explained by psychiatric comorbidity, sleep apnea or RLS. Our results are consistent with previous data on CD and other gastrointestinal diseases suggesting that poor sleep may be a manifestation of CD. Individuals with CD suffer from numerous symptoms that may impair the quality of sleep [2]. Furthermore, even though most CD manifestations typically resolve on a gluten-free diet, some symptoms may persist, in particularly when dietary adherence is poor [32]. Regrettably, we lack dietary data in this study. However, in an earlier validation study off a random sample of individuals with villous atrophy, 15/86 (17%) had poor dietary adherence [13].

Finally, we cannot rule out that the association seen in this study was influenced by surveillance bias with an increased detection of CD in individuals with poor sleep. It is well known that individuals with poor sleep have an increased health care consumption [33], including increased drug use [9]. In addition, studies have shown a mean diagnostic delay of more than five years from onset of CD symptoms to diagnosis [34], a time associated with an increased number of outpatients visits [35] and possibly an increased likelihood of receiving treatment for poor sleep. Regrettably, we lack data on clinical characteristics of those individuals with CD. However, the lack of a time-dependent association between poor sleep and CD diagnosis, argues against surveillance bias as the sole cause of our findings.

## Conclusions

In conclusion, individuals with CD suffer an increased risk of poor sleep, measured as repeated use of prescribed hypnotics, both before and after diagnosis. The excess risk was not influenced by psychiatric comorbidities. Although, we cannot rule out that surveillance bias has contributed to our findings, our results are consistent with earlier research suggesting that poor sleep may be a manifestation of CD.

## Additional files

Additional file 1:
**Anatomical therapeutic chemical codes used to classify hypnotics.**
Additional file 2:
**Anatomical therapeutic chemical codes on drugs typically used for treatment of restless legs syndrome.**
Additional file 3:
**International Classification of Disease (ICD) codes version 8-10.**

## Abbreviations

ATC: Anatomical therapeutic chemical
CD: Celiac disease
CI: Confidence interval
HR: Hazard ratio
IBD: Inflammatory bowel disease
ICD: International Classification of Disease
OR: Odds ratio
RLS: Restless legs syndrome
