# Supplementary material for: Increased use of hypnotics in individuals with celiac disease: a nationwide case-control study
Source: BMC Gastroenterol. 2015 Feb 5;15:10. doi: 10.1186/s12876-015-0236-z (PMC4322544; doi:10.1186/s12876-015-0236-z)
Supplement: Additional file 1: — Anatomical therapeutic chemical codes used to classify hypnotics. [file 12876_2015_236_MOESM1_ESM.doc]

**Additional file 1. Anatomical therapeutic chemical codes used to classify hypnotics.**

| **Type of hypnotic** | **Anatomical therapeutic chemical code** | **Description** |
| --- | --- | --- |
| Benzodiazepine-related drugs | N05CF01  N05CF02  N05CF03 | Zopiklon  Zolpidem  Zaleplon |
| Benzodiazepines | N05CD02  N05CD03  N05CD05 | Nitrazepam  Flunitrazepam  Triazolam |
| Other hypnotics | N05CM02  N05CM06 | Clomethiazole  Propiomazine |
| Melatonin receptor agonists | N05CH01 | Melatonin |
