# Supplementary material for: Increased use of hypnotics in individuals with celiac disease: a nationwide case-control study
Source: BMC Gastroenterol. 2015 Feb 5;15:10. doi: 10.1186/s12876-015-0236-z (PMC4322544; doi:10.1186/s12876-015-0236-z)
Supplement: Additional file 2: — Anatomical therapeutic chemical codes on drugs typically used for treatment of restless legs syndrome. [file 12876_2015_236_MOESM2_ESM.doc]

**Additional file 2. Anatomical therapeutic chemical codes on drugs typically used for treatment of restless legs syndrome.**

| **Type of Drug** | **Anatomical therapeutic chemical code** | **Description** |
| --- | --- | --- |
| Anti-epileptics | N03AX16  N03AX12 | Pregabalain  Gabapentin |
| Dopamine and dopamine derivate | N04BA02 | Levodopa |
| Dopamine agonists | N04BC05  N04BC04  N04BC09 | Pramipexole  Ropinirole  Rotigotin |
