# Supplementary material for: Increased use of hypnotics in individuals with celiac disease: a nationwide case-control study
Source: BMC Gastroenterol. 2015 Feb 5;15:10. doi: 10.1186/s12876-015-0236-z (PMC4322544; doi:10.1186/s12876-015-0236-z)
Supplement: Additional file 3: — International Classification of Disease (ICD) codes version 8-10. [file 12876_2015_236_MOESM3_ESM.doc]

**Additional file 3.** International Classification of Disease (ICD) codes version 8-10

**Psychiatric comorbidity** (including any of the following conditions):

*Depressive disorders*: ICD-8: 296.0, 300.4; ICD-9: 296B, 300E, 311; ICD-10: F32, F33, F34.1, F34.8, F34.9 and F38.1.

*Anxiety or phobic disorders*: ICD-8: 300.0, 300.2; ICD-9: 300A, 300C; ICD-10: F40, F41.

*Psychotic, substance use and personality disorders*: ICD-8: 291, 293, 295-304, 306-308; ICD-9: 291-306, 307, 312-315; ICD-10: F10-58; F60-69, F80-99.

**Epilepsy**: ICD-7: 353 except for 353.2; ICD-8: 345 except for 345.2; ICD-9: 345 except for 345Q; ICD-10: G40.
